# Association of Physical Fitness With the Work Ability of Aging Workers With Physically Demanding Jobs in a University Hospital in Thailand

**DOI:** 10.1016/j.shaw.2024.06.003

**Published:** 2024-06-14

**Authors:** Jidapa Hanvoravongchai, Methasit Laochindawat, Soontorn Supapong, Jate Ratanachina

**Affiliations:** 1 Department of Preventive and Social Medicine, Faculty of Medicine, Chulalongkorn University, Bangkok, Thailand; 2 National Heart and Lung Institute, Faculty of Medicine, Imperial College London, London, United Kingdom

**Keywords:** Aging, Physical fitness, Work ability

## Abstract

**Background:**

A decrease in physical fitness with age can impact work ability. Exploring the role of physical fitness is important for identifying interventions to enhance work ability among aging workers. We sought to determine the association between physical fitness and physically demanding work ability in aging workers.

**Methods:**

We recruited workers aged 45–65 years from eight departments of a university hospital in Bangkok, Thailand. Work ability was assessed using the work ability index (WAI), and physical fitness components were evaluated through various tests. Associations between work ability and physical fitness were examined using a multivariable regression analysis.

**Results:**

The response rate was 46.4% (*n* = 216). The mean WAI score was 41.6. Participants with an overweight or obese body mass index (BMI) had 1.8 (95% CI –3.1, –0.4) points lower WAI scores than did those within a healthy BMI range. A handgrip strength test revealed that participants in the good/very good group had 2.5 (95% CI: 0.6, 4.3) higher WAI scores than did those in the very poor/poor group. For a single-leg stance test, participants in the above-average group had 2.1 (95% CI: 0.7, 3.5) points greater WAI scores than did those in the below-average group. No significant associations were found between work ability and 3-min step, chair stand, or sit-and-reach test scores.

**Conclusion:**

The overall work ability of the participants was good. Body composition, muscle strength, and balance were associated with work ability. Promoting physical fitness is a viable strategy for enhancing work ability among aging workers.

## 1 Introduction

Globally, the age of populations is advancing, resulting in an increased proportion of aging workers within the workforce. According to the International Labour Organization, it is anticipated that by 2030, developed countries will have a 25% proportion of workers aged ≥55 years, while developing countries will experience an increase of approximately 12% [1]. In Thailand, the number of workers aged ≥60 years has increased from 3.2 million in 2011 to 4.7 million in 2020 [2].

With advancing age, workers experience physiological changes in both cardiorespiratory and musculoskeletal fitness that can lead to a decrease in their work ability [3]. Work ability is defined as the balance between the physical and mental capacity of a worker and the demand of their work [4]. Imbalances occur when a worker's physical capacity decreases while the demand of their work remains the same, resulting in health issues such as cardiovascular disease and mental illness, increased work-related accidents, more sick leave, and early exit from work [5]. Several studies have found an association between physical fitness and work ability. Some have found that muscle strength, muscle endurance, and body mass index (BMI) are associated with work ability, while findings on cardiorespiratory fitness are contradictory [[6], [7], [8], [9], [10]].

Changes in both physical fitness and work ability are likely to be more strongly associated with the ability to perform physically demanding jobs. A 30-year longitudinal study in Copenhagen revealed that among workers with physically demanding jobs, those who have good cardiorespiratory fitness, have a lower risk of cardiovascular death than those with moderate or poor fitness [11]. A study in Italy found a greater decrease in work ability with age among manual workers in a heavy industry than among clerical workers [12].

While healthcare workers, including physicians or nurses, are often participants in work ability studies, it is important to recognize workers in supporting departments in healthcare settings. These sometimes-overlooked roles, such as logistics, central sterile supply, laundry, housekeeping, and security, often involve physically demanding tasks. Investigating the association between physical fitness and work ability could help to promote better work ability in this group of aging workers [13].

In Thailand, existing studies on work ability have focused primarily on demographic, social, and work-related factors associated with work ability in workers. One study in a southern province found that advanced age, non-communicable diseases (NCDs), and an unsafe work environment all contribute to poorer work ability [14]. Another study in a rural area identified younger age, university education, and a healthy body mass index (BMI) as factors associated with higher work ability among aging workers [15]. Notably, studies focusing on the association between physical fitness and work ability are lacking. The present study aimed to determine physical fitness, work ability, and associated factors among aging workers with physically demanding jobs at a university teaching hospital in Bangkok, and the association between their physical fitness and work ability.

## 2 Materials and methods

### 2.1 Study design and participants

We conducted a cross-sectional study. The study population included aging workers, aged 45–65 years, at a university hospital in Bangkok, Thailand, who had been working for at least one year. We attempted to recruit 464 worker participants from eight departments whose work included physically demanding tasks: logistics, inventory management, dietetics, central sterile supply, laundry, mechanics, housekeeping, and security. We determined a sample size of 208 based on the reference value established by Nygard et al [7]. To ensure equal opportunity for participation across departments, we opted for a full recruitment strategy rather than sampling, inviting all eligible workers to participate. The study design and its ethical considerations were approved by the Institutional Review Board, Faculty of Medicine, Chulalongkorn University (No. 166/66). The data were collected between August and September 2023.

### 2.2 Inclusion and exclusion criteria

Candidates with neurological problems affecting movement and balance, a recent history of myocardial infarction, or other conditions restricting moderate and heavy exercise, and those with jobs not classified as physically demanding were excluded from the study. Participants presenting with systolic blood pressure exceeding 160 mmHg, diastolic blood pressure exceeding 100 mmHg, or a heart rate exceeding 110 bpm on the day of data collection were excluded from the 3-minute step test and chair stand test. Those who used antiarrhythmic medications were also excluded from the 3-minute step test. In addition, participants with recent upper extremity surgery or acute injuries were excluded from the handgrip strength test, and those with recent back surgery or experiencing back pain were excluded from the sit-and-reach test. All exclusion criteria were assessed by physicians on the day of data collection.

### 2.3 Demographic data collection

The questionnaire was used to collect the demographic characteristics of participants, which included age, sex, educational level (primary, secondary, or higher than secondary school), marital status (single, married, divorced, or widowed), smoking history (never/ever), alcohol consumption (no/yes), leisure-time physical activity (0–1 day/week, 2–4 days/week, and ≥5 days/week), job tenure (years), shift work (no/yes), income per month (THB), and department.

### 2.4 Work ability assessment

The work ability index (WAI) is a structured questionnaire widely used in clinical occupational health research to assess the perceived work abilities of individuals. We assessed the work ability of participants using the Thai version of the WAI [16]. The index covers 7 dimensions, including (1) current work ability compared with the lifetime best, (2) work ability in relation to the demands of the job, and (3) number of current diseases diagnosed by a physician, (4) estimated work impairment due to diseases, (5) sick leave during the past year, (6) own prognosis of work ability 2 years from now, and (7) mental resources. The total scores ranged from 7 to 49 points and were categorized into 4 groups: poor (7 to 27), moderate (28 to 36), good (37 to 43), and excellent (44 to 49) [4].

### 2.5 Physical fitness assessment

After completing the questionnaire, eligible participants completed 6 physical fitness tests administered by physicians, which included BMI measurements, a 3-min step test, a handgrip strength test, a chair stand test, a sit-and-reach test, and a single leg stance test. The physical fitness criteria used in the study are described in the online supplementary material.

BMI was used to assess body composition. The weight and height of the participants were measured, and their BMI was calculated and categorized according to recommendations for people of Asia-Pacific ethnicity (Table S1) [17].

A 3-min step test was used to assess cardiorespiratory fitness. Eligible participants were asked to step up and down from a 30-cm-high box rhythmically for 3 min. The pulse rate was subsequently measured using criteria from the Department of Physical Education, Ministry of Tourism and Sports, Thailand (DPE) (Table S2) [18].

A handgrip strength test was used to assess muscle strength. Participants were asked to hold a handgrip dynamometer (Takei T.K.K.5401 grip-D) with their dominant hand. After standing with their arms abducted 15° from their trunk, the participants were asked to squeeze the dynamometer with maximum effort for 3–5 s. The test was conducted twice with an intervening 30-s rest period. Maximum handgrip strength was measured in kg/kg × body weight (kg/kg BW) using criteria from the study by Samhita et al (Table S3) [19].

A chair-stand test was used to assess muscle endurance. Participants aged 45–59 years were asked to sit and stand on a chair as quickly as possible with their hands folded across their chest for 60 s, while those aged ≥60 years were asked to sit in the same way for 30 s. The number of repetitions within the specified period was measured using criteria from the DPE (Table S4) [18].

A sit-and-reach test was used to assess flexibility. Participants were asked to stretch quickly before sitting on a mat. With a measuring line between their legs and the soles of their feet placed behind the baseline at the 15-inch (38 cm) mark, they were asked to reach forward as far as possible. The test was conducted twice. The farthest distance (inches) that the hands could reach was recorded using criteria from the DPE (Table S5) [18].

**Fig. 1:**
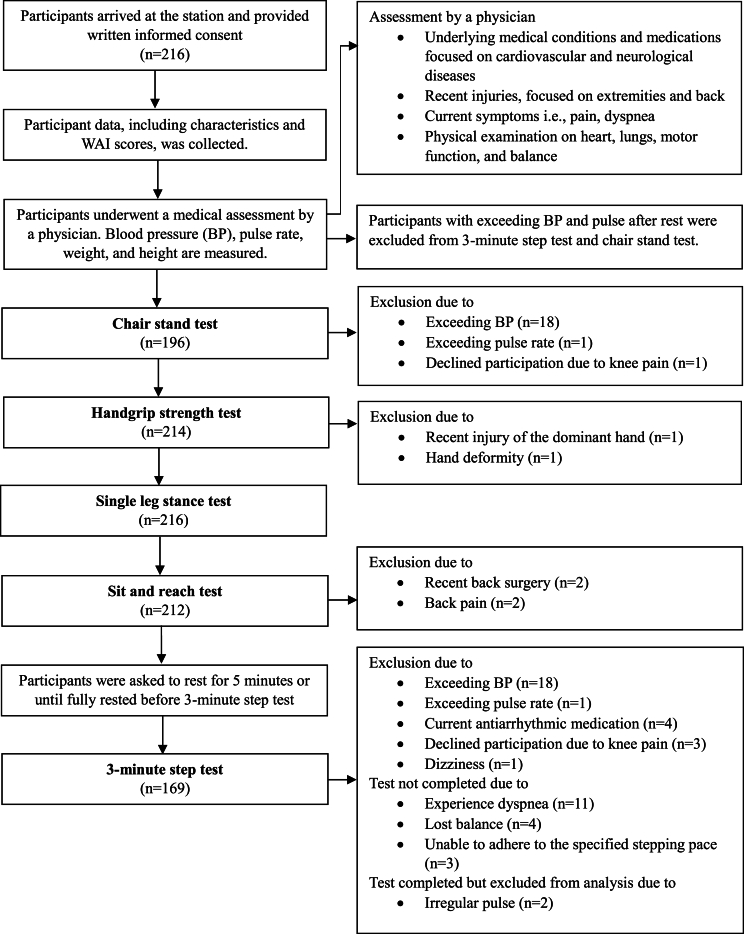
Flow diagram of the data collection process.

A single-leg stance test was used to assess balance. Participants were asked to stand on their dominant foot for as long as possible with the other foot elevated above the ground at the ankle level, their hands folded across their chest, and their eyes looking forward. The test ended when (1) the raised foot touched the ground or moved away from the standing limb, (2) the weight-bearing foot moved from the original position, (3) the hands were not in the original position, or (4) a maximum of 45 s had elapsed. The duration for which the participant could stand on one leg was recorded using criteria from Springer et al (Table S6) [20]. Details of the data collection process are described in Fig. 1.

### 2.6 Statistical analysis

The demographic characteristics are reported as numbers with proportions for categorical variables and as the means and standard deviations for continuous variables. Associations between participant sample characteristics and WAI scores were examined using an independent *t*-test, one-way ANOVA for categorical independent variables, and Pearson correlation for continuous independent variables.

Levels of physical fitness were categorized based on the criteria mentioned above. BMI was categorized into 3 groups: underweight, healthy, and overweight/obese. The results of the 3-min step, handgrip strength, chair stand, and sit-and-reach tests were categorized into 3 groups: very poor/poor, moderate, and good/very good. The single-leg stance test results were categorized as below or above average. Associations between the WAI and physical fitness indicators were examined using multivariable regression analysis. The analysis was presented in three models. Model 1 represents the unadjusted model, Model 2 includes adjustments for sex and age, and Model 3 is fully adjusted for sex, age, engagement in shiftwork, job tenure, and work departments. Sex and age were strongly associated with physical fitness, and previous studies have also shown associations with work ability [21,22]. Shiftwork and job tenure were found to be associated with work ability in this study. We also included work departments in the model due to each department's varying physical work demands, which could affect both physical fitness and work ability.

The significance level for all analyses was set at *p* < 0.05. All the statistical analyses were performed using STATA statistical software, version 17.0 (StataCorp).

## 3 Results

### 3.1 Study population characteristics

The overall response rate was 46.6% (*n* = 216) (Table S7). The characteristics of the participants are shown in Table 1. The mean age was 52.0 (SD 4.7) years (range 45–65 years), and 64.8% were female. Most participants had a secondary school education level (51.4%), were married (60.0%), were nonsmokers (85.2%), and abstained from drinking alcohol (73.6%). The majority reported engaging in leisure-time physical activities of 0–1 day/week (61.9%).

**Table 1:** Characteristics of the study participants (*n* = 216)

Characteristics	Total (*n* = 216)	*p*
**Age (years)**, mean (SD)	52.0 (4.7)	0.1
**Women,***n* (%)	140 (64.8)	0.6
**Educational level,***n* (%)		0.6
Primary school	67 (31.3)	
Secondary school	110 (51.4)	
Higher than secondary school	37 (17.3)	
**Marital status**, *n* (%)		0.2
Single, divorced or widowed	86 (40.0)	
Married	129 (60.0)	
**Smoking history**, *n* (%)		0.1
Never	184 (85.2)	
Ever	32 (14.8)	
**Alcohol drinking**, *n* (%)		0.3
No	159 (73.6)	
Yes	57 (26.4)	
**Leisure-time physical activity (day/week)**, *n* (%)		0.4
0–1	130 (61.9)	
2−4	60 (28.6)	
≥5	20 (9.5)	
**Job tenure (years)**, mean (SD)	22.7 (11.0)	0.05∗
**Shift work**, *n* (%)		0.03∗
No	119 (54.9)	
Yes	97 (45.1)	
**Income per month (THB)**†, *n* (%)		0.5
<15,000	17 (7.9)	
≥15,000	199 (92.1)	
**Department**, *n* (%)		0.7
Logistics	81 (37.5)	
Inventory management	9 (4.2)	
Dietetics	27 (12.5)	
Central sterile supply	17 (7.9)	
Laundry	36 (16.7)	
Mechanics	18 (8.3)	
Housekeeping	18 (8.3)	
Security	10 (4.6)	

∗ *p* < 0.05.

† 15,000 THB = 411 USD on 30 September 2023 (The Bank of Thailand).

For work-related factors, the mean job tenure of the participants was 22.7 (SD 11.0) years (range 1–45 years), 54.9% reported engaging in shiftwork, and 92.1% reported monthly income >15,000 THB (411 USD).

### 3.2 Work ability index

The mean WAI score was 41.6 (SD 5.0). No significant differences in scores were observed between the various departments. The department with the highest mean WAI score was laundry, with a mean score of 42.89, while the department with the lowest was in the central sterile supply, with a mean score of 40.21. When WAI scores were categorized into 4 groups, 32.9% reported excellent, 56.0% good, 10.2% moderate, and 0.9% poor work ability. Table 2 presents the distribution of scores for each WAI dimension. Bivariate analysis of each demographic characteristic revealed that engagement in shiftwork was positively associated with the WAI score (*p* < 0.05) and job tenure was inversely associated with the WAI score (*p* < 0.05).

**Table 2:** WAI scores of the study participants distributed by dimensions (*n* = 216)

Dimensions of WAI	Score *n* (%)										
	0	1	2	3	4	5	6	7	8	9	10
1. Current work ability compared with the lifetime best	0 (0.0)	0 (0.0)	0 (0.0)	0 (0.0)	0 (0.0)	9 (4.2)	9 (4.2)	27 (12.5)	67 (31.0)	46 (21.3)	58 (26.9)
2. Work ability in relation to the demands of the job			0 (0.0)	0 (0.0)	1 (0.5)	2 (0.9)	69 (31.9)	35 (16.2)	55 (25.5)	30 (13.4)	24 (11.1)
3. Number of current diseases diagnosed by a physician		4 (1.9)	6 (2.8)	14 (6.5)	49 (22.7)	51 (23.6)		92 (42.6)			
4. Estimated work impairment due to diseases		0 (0.0)	1 (0.5)	1 (0.5)	3 (1.4)	64 (29.6)	147 (68.1)				
5. Sick leave during the past year		1 (0.5)	13 (6.0)	21 (9.7)	59 (27.3)	122 (56.5)					
6. Own prognosis of work ability 2 years from now		4 (1.9)			24 (11.1)			188 (87.0)			
7. Mental resources		0 (0.0)	14 (6.5)	46 (21.3)	156 (72.2)						

WAI, work ability index.

### 3.3 Physical fitness assessment

Table S8 presents all the physical fitness test results. The BMIs of all 216 participants were measured. The mean BMI was 26.1 (SD 4.4) kg/m^2^. Most participants were classified as overweight/obese (74.1%), while only 23.6% fell into the healthy BMI range, and 2.3% were classified as underweight.

Of the 216 participants, 169 eligible participants completed the 3-minute step test. The mean pulse rate after the test was 130.5 (SD 17.3) bpm, with 68.1% classified in the very poor/poor group.

The handgrip strength test was performed by 214 participants. The mean handgrip strength was 0.5 (SD 0.1) kg/kg BW. Most of the participants were in the moderate strength group (61.7%).

The chair stand test was performed by 196 participants, of whom 184 participants aged <60 years performed the 60-s chair stand test and 12 aged ≥60 years performed the 30-s chair stand test. For the 60-s chair stand test, the mean number of repetitions was 32.6 (SD 10.0) times/min; for the 30-s chair stand test, the mean number of repetitions was 18.0 (SD 5.3) times/min. Most participants scores fell into the good/very good group (60.2%).

For the sit-and-reach test, 212 participants were assessed.The mean distance that participants could bend forward was 15.8 (SD 3.9) inches. Most participant results were in the moderate group (55.2%).

All 216 participants performed the single-leg stance test for a mean duration of 28.7 (SD 12.3) s. After accounting for their sex and age group, 77.8% of the participants performed the test with an above-average duration. Reasons for exclusions for each physical fitness test are shown in Fig. 1.

### 3.4 Associations between work ability and physical fitness

Table 3 presents the associations between physical fitness and work ability. According to the fully adjusted model, participants classified as overweight/obese had 1.8 (95% CI –3.1, –0.4) points lower WAI scores than did those within a healthy BMI range. For the handgrip strength test, participants classified as moderate had 1.6 (95% CI 0.1, 3.1) points higher WAI scores than did those in the very poor/poor group, while participants classified as good/very good had 2.5 (95% CI 0.6, 4.3) points higher WAI scores than those in the very poor/poor group. Participants in the above-average group of single-leg stance test results had 2.1 (95% CI 0.7, 3.5) points greater WAI scores than did those in the below-average group. Similar results were observed in other models. In models 1 and 2, participants classified as overweight had 1.5 points lower WAI scores compared to those within a healthy BMI range. Participants classified as good/very good in the handgrip strength test had 2.0 (95% CI 0.3, 3.8) and 2.4 (95% CI 0.6, 4.2) points higher WAI scores than those in the very poor/poor group in models 1 and 2, respectively. Participants with above-average single-leg stance test results scored 2.0 (95% CI 0.7, 3.3) points and 1.8 (95% CI 0.5, 3.1) points higher than those in the below-average group in models 1 and 2, respectively. No significant associations were found between the 3-minute step test, chair stand test, sit and reach test, and work ability across the three models.

**Table 3:** Associations between work ability and physical fitness (*n* = 216)

Physical fitness indicators	Physical fitness levels	*n* (%)	Model 1†		Model 2‡		Model 3§	
			β	95% CI	β	95% CI	β	95% CI
BMI	Healthy	51 (23.6)	Ref		Ref		Ref	
	Underweight	5 (2.3)	−2.2	−5.9, 1.6	−2.4	−6.2, 1.3	−2.8	−6.6, 1.0
	Overweight/obese	160 (74.1)	−1.5∗	−2.8, −0.2	−1.5∗	−6.2, −2.8	−1.8∗	−3.1, −0.4
3-minute step	Very poor/poor	115 (68.1)	Ref		Ref		Ref	
	Moderate	36 (21.3)	0.6	−0.9, 2.2	0.5	−1.1, 2.1	0.6	−1.1, 2.2
	Good/very good	18 (10.7)	0.9	−1.1, 3.0	1.2	−0.9, 3.3	1.3	−1.0, 3.5
Handgrip strength	Very poor/poor	39 (18.2)	Ref		Ref		Ref	
	Moderate	132 (61.7)	1.3	−0.1, 2.8	1.6∗	0.1, 3.1	1.6∗	0.1, 3.1
	Good/very good	43 (20.1)	2.0∗	0.3, 3.8	2.4∗	0.6, 4.2	2.5∗	0.6, 4.3
Chair stand	Very poor/poor	37 (18.9)	Ref		Ref		Ref	
	Moderate	41 (20.9)	−0.8	−2.0, 1.8	−0.7	−2.0, 1.8	−0.1	−2.0, 1.9
	Good/very good	118 (60.2)	−0.3	−1.6, 1.5	−0.1	−1.5, 1.7	−0.1	−1.7, 1.6
Sit and reach	Very poor/poor	34 (16.0)	Ref		Ref		Ref	
	Moderate	117 (55.2)	−1.4	−2.9, 0.2	−1.4	−2.9, 0.2	−1.2	−2.8, 0.4
	Good/very good	61 (28.8)	0.3	−1.4, 2.0	0.5	−1.2, 2.2	0.5	−1.3, 2.3
Single leg stance	Below average	48 (22.2)	Ref		Ref		Ref	
	Above average	175 (77.8)	2.0∗	0.7, 3.3	1.8∗	0.5, 3.1	2.1∗	0.7, 3.5

BMI, body mass index.

∗ *p* < 0.05.

† No adjustment.

‡ Adjusted for gender and age.

§ Adjusted for gender, age, engaging in shiftwork, job tenure and work departments.

## 4 Discussion

The WAI score of the study population sample was generally good (a mean WAI score of 41.6), and work ability was found to be associated with body composition, muscle strength (the handgrip strength test), and balance (the single leg stance test) among aging workers. The mean WAI scores reported from previous studies in similar populations were varied, with some studies indicating lower scores and others indicating higher scores. Sociodemographic and work-related factors associated with WAI scores also showed variability. For instance, a cohort study by Martinez et al reported that high mental stress and low income were associated with decreased work ability among hospital workers in Brazil [23]. Whereas, in our study, the majority of participants reported the highest level of mental fitness and appropriate income levels. Andrade et al reported the older age group (50–60 years old) as a major factor associated with low work ability among those with physically demanding work in a hospital [24], while our study found no significant association. Nevertheless, in the present study, we found a significant association between work ability and job tenure, which was aligned with age.

The study revealed an association between BMI categories and work ability, with both the underweight and overweight/obese groups exhibiting lower mean WAI scores than did those within a healthy BMI range. This finding is consistent with previous studies that have shown an association between higher BMI categories and poor work ability [8,9]. This finding is unsurprising, given the well-established evidence linking higher BMI to NCDs [25]. Individuals with NCDs tend to take more sick leave than those without underlying conditions [26], and both current disease and sick leave are dimensions considered in the work ability index.

We found that handgrip strength and single-leg stance performance categories were associated with work ability. These findings are consistent with previous studies that have similarly demonstrated an association between muscle strength, balance, and work ability. The level of muscle strength is positively associated with the ability to work. In addition, several tasks involves lifting and pushing objects require not only muscle strength but also demand a balance for smooth movement and the prevention of falls [27]. The physically demanding nature of the jobs of our study participants provides a plausible explanation for these associations. The daily tasks of participants in most departments require muscle strength, especially in the upper extremities. For instance, individuals in the logistics department require upper extremity strength for lifting objects and pushing carts or wheelchairs; those in the dietetics department require upper extremity strength for cooking and preparing food ingredients; and those in the laundry departments require upper extremity strength for ironing and carrying clothes.

Interestingly, in our present study, a 3-min step test representing cardiorespiratory fitness was not significantly associated with WAI scores. Previous studies have reported contradictory findings regarding the association between cardiorespiratory fitness and work ability. For instance, a study by Nygard et al on aging municipal employees, which used a submaximal cycle-ergometer test for cardiorespiratory fitness assessment, found no association between VO_2_ max and WAI scores [7]. Conversely, a study by Sörensen et al, evaluated municipal employees using a maximal bicycle ergometer test and a 2-km walking test and found significant associations [7]. Anbazhagan et al studied aging tea plantation workers in India, using the 3-min step test, found a significant association [28]. The varied findings can be explained by the use of different tests used to assess cardiorespiratory fitness, diverse demographics, and diverse results of fitness levels among the study participants. Notably, most of the participants in the study by Sörenson were in a very poor/poor group, while in the study by Anbazhagan et al, the majority were in a good/very good group [26]. The likelihood that physically demanding jobs impose more stress on the musculoskeletal system than on the cardiorespiratory system may also explain the varied findings.

We did not find associations between work ability and muscle endurance or flexibility. Previous studies examining the associations between muscle endurance and work ability have reported mixed results, depending on the muscle groups assessed. A study by Suorsa et al found associations between muscle endurance and work ability when using chair stand and push-up tests but not sit-up tests [6]. Similarly, a study among physical therapists by Ezzatva et al reported associations between back-muscle endurance and work ability, but no associations were found with push-up tests [29]. These varied findings may result from the different muscles involved in various job tasks. Future studies focusing on different muscle groups may provide additional insights into the association between muscle endurance and work ability. To our knowledge, no association between flexibility and work ability has been published to date. However, Han et al showed that hamstring stretching, which enhances flexibility, was associated with improved work ability in those who work standing, emphasizing the importance of further investigations into this topic [30].

The strengths of the present study include the wide range of physical fitness tests covering 5 health-related physical fitness components, namely body composition, cardiorespiratory fitness, muscle strength, muscle endurance, and flexibility, including balance assessment. The tests were selected based on their safety for aging people, their ease of administration without the need for extensive equipment, time efficiency, and the presence of criteria to categorize results for the study population. The on-site administration of questionnaires minimized missing data, as the questionnaire was double-checked by our research team. There were no missing data for the WAI questionnaire, allowing the analysis of data from all 216 participants. Nevertheless, limitations remain, including its cross-sectional study design, so that a causal relationship cannot be assumed. Daily total physical activity was not measured using accelerators; instead, we used the questionnaire to collect data on leisure-time physical activity, and the data were analyzed by adjusting for participants' work departments under the assumption that individuals within the same department experience similar daily physical activities. Physical fitness tests were conducted during working hours, with participants in work uniforms, and some uniforms may not have been suitable for the exercises. For instance, tight uniforms could make it difficult for participants to bend forward during the sit-and-reach test. Furthermore, most criteria used to categorize the results of physical fitness tests are specific to the Thai population at a single institution, limiting the extrapolation of the results to populations of other ethnicities or at different locations.

Our findings suggest that several physical fitness components, including body composition, muscle strength, and balance, are associated with work ability in aging workers. Therefore, interventions to promote physical fitness in the workplace are recommended. These interventions could include health education, counseling, or structured training sessions incorporating resistance training and balance exercises. Physical fitness evaluation and incentives could be employed to motivate aging workers to improve their physical fitness.

Under circumstances where resources such as equipment, personnel, and time are not limited, more complex physical fitness tests can be conducted for more precise results. For example, a maximal treadmill or bicycle ergometer can be used to assess cardiorespiratory fitness, bioelectrical impedance analysis can be used to assess body composition, and the mini-BEST (mini-Balance Evaluation System Test) can be used to assess both static and dynamic balance. Additionally, physical fitness assessments for specific body parts, such as push-ups and sit-up tests can be conducted to assess muscle endurance for the upper extremities and abdominal muscles, respectively. Similar studies can be conducted for specific occupations with a focus on the fitness components and body parts that are relevant to the job tasks. Finally, we recommend longitudinal studies to examine the effects of physical fitness training on the work ability of aging workers.

### 4.1 Conclusions

Promoting physical fitness is a strategy that may improve the work ability of aging workers with physically demanding jobs.

## CRediT authorship contribution statement

**Jidapa Hanvoravongchai:** Conceptualization, Data curation, Formal analysis, Investigation, Methodology, Visualization, Writing – original draft, Writing – review & editing. **Methasit Laochindawat:** Conceptualization, Data curation, Investigation, Writing – review & editing. **Soontorn Supapong:** Conceptualization, Funding acquisition, Writing – review & editing, Methodology. **Jate Ratanachina:** Conceptualization, Formal analysis, Methodology, Supervision, Writing – original draft, Writing – review & editing.

## Conflicts of interest

The authors certify that they have NO affiliations with or involvement in any organization or entity with any financial interest (such as honoraria; educational grants; participation in speakers' bureaus; membership, employment, consultancies, stock ownership, or other equity interest; and expert testimony or patent-licensing arrangements), or non-financial interest (such as personal or professional relationships, affiliations, knowledge or beliefs) in the subject matter or materials discussed in this manuscript.
