# Supplementary material for: Association of Physical Fitness With the Work Ability of Aging Workers With Physically Demanding Jobs in a University Hospital in Thailand
Source: Saf Health Work. 2024 Jun 14;15(3):345–51. doi: 10.1016/j.shaw.2024.06.003 (PMC11410491; doi:10.1016/j.shaw.2024.06.003)
Supplement: Multimedia component 1 [file mmc1.docx]

**Online supplementary material**

**Table S1** Body mass index criteria

| **Category** | **Body mass index (kg/m^2^)** |
| --- | --- |
| Underweight | < 18.5 |
| Normal | 18.5-22.9 |
| Overweight | 23-24.9 |
| Obese I | 25-29.9 |
| Obese II | ≥30 |

Source: Pan WH, Yeh WT. How to define obesity? Evidence-based multiple action points for public awareness, screening, and treatment: an extension of Asian-Pacific recommendations. Asia Pac J Clin Nutr 2008; 17:370-4.

**Table S2** 3-minute step test criteria

**Criteria for men**

| **Age category**  **(years)** | **Pulse rate after performing 3-minute step test (beats/min: bpm)** | | | | |
| --- | --- | --- | --- | --- | --- |
|  | **Very good** | **Good** | **Moderate** | **Poor** | **Very poor** |
| 20-29 | ≤88 | 89-97 | 98-116 | 117-125 | ≥126 |
| 30-39 | ≤93 | 94-102 | 103-121 | 122-130 | ≥131 |
| 40-49 | ≤93 | 94-102 | 103-121 | 122-130 | ≥131 |
| 50-59 | ≤91 | 92-100 | 101-119 | 120-128 | ≥129 |
| 60-69 | ≤89 | 90-97 | 98-114 | 115-122 | ≥123 |

**Criteria for women**

| **Age category**  **(years)** | **Pulse rate after performing 3-minute step test (beats/min: bpm)** | | | | |
| --- | --- | --- | --- | --- | --- |
|  | **Very good** | **Good** | **Moderate** | **Poor** | **Very poor** |
| 20-29 | ≤107 | 108-115 | 116-132 | 133-140 | ≥141 |
| 30-39 | ≤103 | 104-111 | 112-128 | 129-136 | ≥137 |
| 40-49 | ≤102 | 103-110 | 111-127 | 128-135 | ≥136 |
| 50-59 | ≤100 | 101-108 | 109-125 | 126-133 | ≥134 |
| 60-69 | ≤101 | 102-109 | 110-126 | 127-134 | ≥135 |

Source: Department of Physical Education. Manual of tests and physical fitness standards for children, youth, and Thai citizens. Bangkok: Word Expert Co Ltd.; 2019.

**Table S3** Handgrip strength test criteria

**Criteria for men**

| **Age category**  **(years)** | **Handgrip strength (kg/kg body weight: kg/kgBW)** | | | | |
| --- | --- | --- | --- | --- | --- |
|  | **Very good** | **Good** | **Moderate** | **Poor** | **Very poor** |
| 20-29 | ≥1.05 | 0.83-1.04 | 0.61-0.82 | 0.37-0.60 | ≤0.36 |
| 30-39 | ≥0.92 | 0.74-0.91 | 0.54-0.73 | 0.26-0.53 | ≤0.25 |
| 40-49 | ≥0.84 | 0.68-0.83 | 0.52-0.67 | 0.34-0.51 | ≤0.33 |
| 50-59 | ≥0.84 | 0.66-0.83 | 0.48-0.65 | 0.27-0.47 | ≤0.26 |
| 60-69 | ≥0.66 | 0.54-0.65 | 0.36-0.53 | 0.24-0.35 | ≤0.23 |

**Criteria for women**

| **Age category**  **(years)** | **Handgrip strength (kg/kg body weight: kg/kgBW)** | | | | |
| --- | --- | --- | --- | --- | --- |
|  | **Very good** | **Good** | **Moderate** | **Poor** | **Very poor** |
| 20-29 | ≥0.78 | 0.58-0.77 | 0.38-0.77 | 0.15-0.38 | ≤0.14 |
| 30-39 | ≥0.63 | 0.51-0.62 | 0.39-0.50 | 0.27-0.38 | ≤0.26 |
| 40-49 | ≥0.62 | 0.50-0.61 | 0.37-0.49 | 0.25-0.36 | ≤0.24 |
| 50-59 | ≥0.64 | 0.48-0.63 | 0.31-0.47 | 0.19-0.30 | ≤0.18 |
| 60-69 | ≥0.55 | 0.41-0.54 | 0.27-0.40 | 0.14-0.26 | ≤0.13 |

Source: Samahito S, Tientong A, Pattaropat W, Reungthai R, Sriyaphai A. Physical fitness standards for Thai citizens. Kasetsart Educ Rev 2005;2:27-40.

**Table S4** Chair stand test criteria

**Criteria for 60-second chair stand test for men aged 19-59 years**

| **Age category**  **(years)** | **Number of repetitions (times/min)** | | | | |
| --- | --- | --- | --- | --- | --- |
|  | **Very good** | **Good** | **Moderate** | **Poor** | **Very poor** |
| 19-24 | ≥54 | 46-53 | 39-45 | 32-38 | ≤31 |
| 25-29 | ≥53 | 47-52 | 39-46 | 31-38 | ≤30 |
| 30-34 | ≥52 | 43-51 | 34-42 | 26-33 | ≤25 |
| 35-39 | ≥50 | 42-49 | 34-41 | 25-33 | ≤24 |
| 40-44 | ≥48 | 41-47 | 33-40 | 25-32 | ≤24 |
| 45-49 | ≥46 | 38-45 | 30-37 | 23-29 | ≤22 |
| 50-54 | ≥43 | 36-42 | 28-35 | 20-27 | ≤19 |
| 55-59 | ≥41 | 34-40 | 26-33 | 19-25 | ≤18 |

**Criteria for 60-second chair stand test for women aged 19-59 years**

| **Age category**  **(years)** | **Number of repetitions (times/min)** | | | | |
| --- | --- | --- | --- | --- | --- |
|  | **Very good** | **Good** | **Moderate** | **Poor** | **Very poor** |
| 19-24 | ≥49 | 41-48 | 33-40 | 25-32 | ≤24 |
| 25-29 | ≥46 | 39-45 | 31-38 | 24-30 | ≤23 |
| 30-34 | ≥45 | 38-44 | 30-37 | 23-29 | ≤22 |
| 35-39 | ≥43 | 36-42 | 29-35 | 22-28 | ≤21 |
| 40-44 | ≥41 | 34-40 | 27-33 | 21-26 | ≤20 |
| 45-49 | ≥36 | 29-35 | 23-28 | 17-22 | ≤16 |
| 50-54 | ≥31 | 25-30 | 19-24 | 13-18 | ≤12 |
| 55-59 | ≥30 | 24-29 | 18-23 | 12-17 | ≤11 |

**Criteria for 30-second chair stand test for men aged 60 years and above**

| **Age category**  **(years)** | **Number of repetitions (times/min)** | | | | |
| --- | --- | --- | --- | --- | --- |
|  | **Very good** | **Good** | **Moderate** | **Poor** | **Very poor** |
| 60-64 | ≥33 | 28-32 | 23-37 | 18-22 | ≤17 |
| 65-69 | ≥31 | 26-30 | 21-25 | 15-20 | ≤14 |

**Criteria for 30-second chair stand test for women aged 60 years and above**

| **Age category**  **(years)** | **Number of repetitions (times/min)** | | | | |
| --- | --- | --- | --- | --- | --- |
|  | **Very good** | **Good** | **Moderate** | **Poor** | **Very poor** |
| 60-64 | ≥29 | 26-28 | 21-25 | 16-20 | ≤15 |
| 65-69 | ≥26 | 22-25 | 17-21 | 13-16 | ≤12 |

Source: Department of Physical Education. Manual of tests and physical fitness standards for children, youth, and Thai citizens. Bangkok: Word Expert Co Ltd.; 2019.

**Table S5** Sit and reach test criteria

**Criteria for men**

| **Age category**  **(years)** | **Furthest distance reached (inches)** | | | | |
| --- | --- | --- | --- | --- | --- |
|  | **Very good** | **Good** | **Moderate** | **Poor** | **Very poor** |
| 20-29 | ≥22 | 20-21 | 15-19 | 13-14 | ≤12 |
| 30-39 | ≥20 | 18-19 | 13-17 | 11-12 | ≤10 |
| 40-49 | ≥21 | 18-20 | 11-17 | 8-10 | ≤7 |
| 50-59 | ≥18 | 16-17 | 11-15 | 9-10 | ≤8 |
| 60-69 | ≥19 | 16-18 | 9-15 | 6-8 | ≤5 |

**Criteria for women**

| **Age category**  **(years)** | **Furthest distance reached (inches)** | | | | |
| --- | --- | --- | --- | --- | --- |
|  | **Very good** | **Good** | **Moderate** | **Poor** | **Very poor** |
| 20-29 | ≥21 | 19-20 | 14-18 | 12-13 | ≤11 |
| 30-39 | ≥24 | 21-23 | 14-20 | 11-13 | ≤10 |
| 40-49 | ≥23 | 20-22 | 13-19 | 10-12 | ≤9 |
| 50-59 | ≥21 | 19-20 | 14-18 | 12-13 | ≤11 |
| 60-69 | ≥21 | 19-20 | 14-18 | 12-13 | ≤11 |

Source: Department of Physical Education. Manual of tests and physical fitness standards for children, youth, and Thai citizens. Bangkok: Word Expert Co Ltd.; 2019.

**Table S6** Single leg stance test criteria

**Criteria for men**

| **Age category (years)** | **Average time on single leg stance (sec)** |
| --- | --- |
| 18-39 | 43.2 |
| 40-49 | 40.1 |
| 50-59 | 38.1 |
| 60-69 | 28.7 |
| 70-79 | 18.3 |

**Criteria for women**

| **Age category (years)** | **Average time on single leg stance (sec)** |
| --- | --- |
| 18-39 | 43.5 |
| 40-49 | 40.4 |
| 50-59 | 36.0 |
| 60-69 | 25.1 |
| 70-79 | 11.3 |

Source: Springer BA, Marin R, Cyhan T, Roberts H, Gill NW. Normative values for the unipedal stance test with eyes open and closed. J Geriatr Phys Ther 2007;30:8-15.

**Table S7** Number of participants from each department

| **Department** | **Total Number** | **Number of participants** | **Participation rate (%)** |
| --- | --- | --- | --- |
| Logistics | 156 | 81 | 51.9 |
| Inventory management | 11 | 9 | 81.8 |
| Dietetics | 48 | 27 | 56.3 |
| Central sterile supply | 36 | 17 | 47.2 |
| Laundry | 47 | 36 | 76.6 |
| Mechanics | 28 | 17 | 60.7 |
| Housekeeping | 121 | 18 | 14.9 |
| Security | 17 | 10 | 58.8 |
| **Total** | **464** | **216** | **46.6** |

**Table S8** Physical fitness of the study participants (n=216)

| **Physical fitness indicators** | **Mean (SD)** | **Categorized using standard criteria**  **n (%)** | | **Categorized into three groups**  **n (%)** | |
| --- | --- | --- | --- | --- | --- |
| **BMI^a^**  (n=216) | 26.1 (4.4) kg/m^2^ | Healthy | 51 (23.6) | Healthy | 51 (23.6) |
|  |  | Underweight | 5 (2.3) | Underweight | 5 (2.3) |
|  |  | Overweight | 40 (18.5) | Overweight/obese | 160 (74.1) |
|  |  | Obese 1 | 80 (37.0) |  |  |
|  |  | Obese 2 | 40 (18.5) |  |  |
| **3-minute step test^b^**  (n=169) | 130.5 (17.3) bpm | Very poor | 82 (48.5) | Very poor/poor | 115 (68.1) |
|  |  | Poor | 33 (19.5) | Moderate | 36 (21.3) |
|  |  | Moderate | 36 (21.3) | Good/very good | 18 (10.7) |
|  |  | Good | 10 (5.9) |  |  |
|  |  | Very good | 8 (4.7) |  |  |
| **Handgrip strength test^c^**  (n=214) | 0.5 (0.1)  kg/kgBW | Very poor | 4 (1.9) | Very poor/poor | 39 (18.2) |
|  |  | Poor | 35 (16.4) | Moderate | 132 (61.7) |
|  |  | Moderate | 132 (61.7) | Good/very good | 43 (20.1) |
|  |  | Good | 39 (18.2) |  |  |
|  |  | Very good | 4 (1.9) |  |  |
| **Chair stand test^d^**  (n=196)  **60 s Chair stand test**  (age <60 years, n=184)  **30 s Chair stand test**  (age ≥60 years, n=12) | 32.6 (10.0) per min  18.0 (5.3) per min | Very poor | 6 (3.1) | Very poor/poor | 37 (18.9) |
|  |  | Poor | 31 (15.8) | Moderate | 41 (20.9) |
|  |  | Moderate | 41 (20.9) | Good/very good | 118 (60.2) |
|  |  | Good | 58 (29.6) |  |  |
|  |  | Very good | 60 (30.6) |  |  |
| **Sit and reach test^e^**  (n=212) | 15.8 (3.9) inches | Very poor | 11 (5.2) | Very poor/poor | 34 (16.0) |
|  |  | Poor | 23 (10.9) | Moderate | 117 (55.2) |
|  |  | Moderate | 117 (55.2) | Good/very good | 61 (28.8) |
|  |  | Good | 35 (16.5) |  |  |
|  |  | Very good | 26 (12.3) |  |  |
| **Single leg stance test^f^**  (n=216) | 38.7 (12.3) sec | Below average | 48 (22.2) |  |  |
|  |  | Above average | 175 (77.8) |  |  |

^a^ BMI results were categorized using Asian-Pacific population criteria.

^b, d, e^ 3-minute step test, chair stand test and sit and reach test results were categorized using criteria by the Department of Physical Education, Ministry of Tourism and Sports, Thailand.

^c^ Handgrip strength test results were categorized using criteria by Samahito *et al.*

^f^ Single leg stance test results were categorized using criteria by Springer *et al.*
